# The potential of lactoferrin as antiviral and immune-modulating agent in viral infectious diseases

**DOI:** 10.3389/fimmu.2024.1402135

**Published:** 2024-11-15

**Authors:** Furkan Eker, Hatice Duman, Melih Ertürk, Sercan Karav

**Affiliations:** ^1^ Department of Molecular Biology and Genetics, Çanakkale Onsekiz Mart University, Çanakkale, Türkiye; ^2^ Uluova Dairy, Canakkale, Türkiye

**Keywords:** lactoferrin, heparan sulfate proteoglycans, viral interference, receptor competition, immune modulating, viral infectious diseases

## Abstract

Emerging infectious diseases are caused by unpredictable viruses with the dangerous potential to trigger global pandemics. These viruses typically initiate infection by utilizing the anionic structures of host cell surface receptors to gain entry. Lactoferrin (Lf) is a multifunctional glycoprotein with multiple properties such as antiviral, anti-inflammatory and antioxidant activities. Due to its cationic structure, Lf naturally interacts with certain host cell receptors, such as heparan sulfate proteoglycans, as well as viral particles and other receptors that are targeted by viruses. Therefore, Lf may interfere with virus-host cell interactions by acting as a receptor competitor for viruses. Herein we summarize studies in which this competition was investigated with SARS-CoV-2, Zika, Dengue, Hepatitis and Influenza viruses *in vitro*. These studies have demonstrated not only Lf’s competitive properties, but also its potential intracellular impact on host cells, such as enhancing cell survival and reducing infection efficiency by inhibiting certain viral enzymes. In addition, the immunomodulatory effect of Lf is highlighted, as it can influence the activity of specific immune cells and regulate cytokine release, thereby enhancing the host’s response to viral infections. Collectively, these properties promote the potential of Lf as a promising candidate for research in viral infectious diseases.

## 1 Introduction

A viral infection occurs when a virus enters a host and begins to multiply, which can potentially lead to disease. The Institute of Medicine (USA) defines emerging infectious diseases as infections that are new, re-emerging, or drug-resistant and frequently infect humans (1). In other words, viral infections expected to become more common and spread rapidly over a wide geographic area are referred to as “emerging infectious diseases” (2). The unpredictable nature of these viruses can dangerously lead to regional or global pandemics, as seen in the most recent example: severe acute respiratory syndrome coronavirus 2 (SARS-CoV-2).

Studying host-virus interactions is essential in viral research, especially given the risk of these infectious diseases emerging. The literature includes many antiviral agents that demonstrate extensive antiviral and therapeutic activities. However, a considerable number of these agents have not been fully analyzed to understand their effects on host immune response or the molecular interactions between host and virus cells. Therefore, analyzing antiviral agents to understand and enhance the host immune response, while preventing viral infections from a molecular perspective, is crucial for studying viral infectious diseases.

Lactoferrin (Lf) is an 80-kDa iron-binding glycoprotein composed of a chain of 690 amino acid residues (3). It possesses diverse multifunctional properties, including antibacterial, antiviral, anti-inflammatory, antioxidant, iron regulatory, and anticarcinogenic activities (4–7). Lf is abundant in milk (particularly in cattle and humans) and is also present in saliva, on mucosal surfaces, and in seminal fluid (4). It has a high affinity for iron, even at low pH values, especially compared to transferrin (8). This strong iron-binding affinity contributes to Lf’s antimicrobial activity by preventing pathogenic bacteria from accessing the iron ions which is considered essential for their growth and survival. On top of that, Lf can modulate iron accumulation under certain conditions.

**Figure 1:**
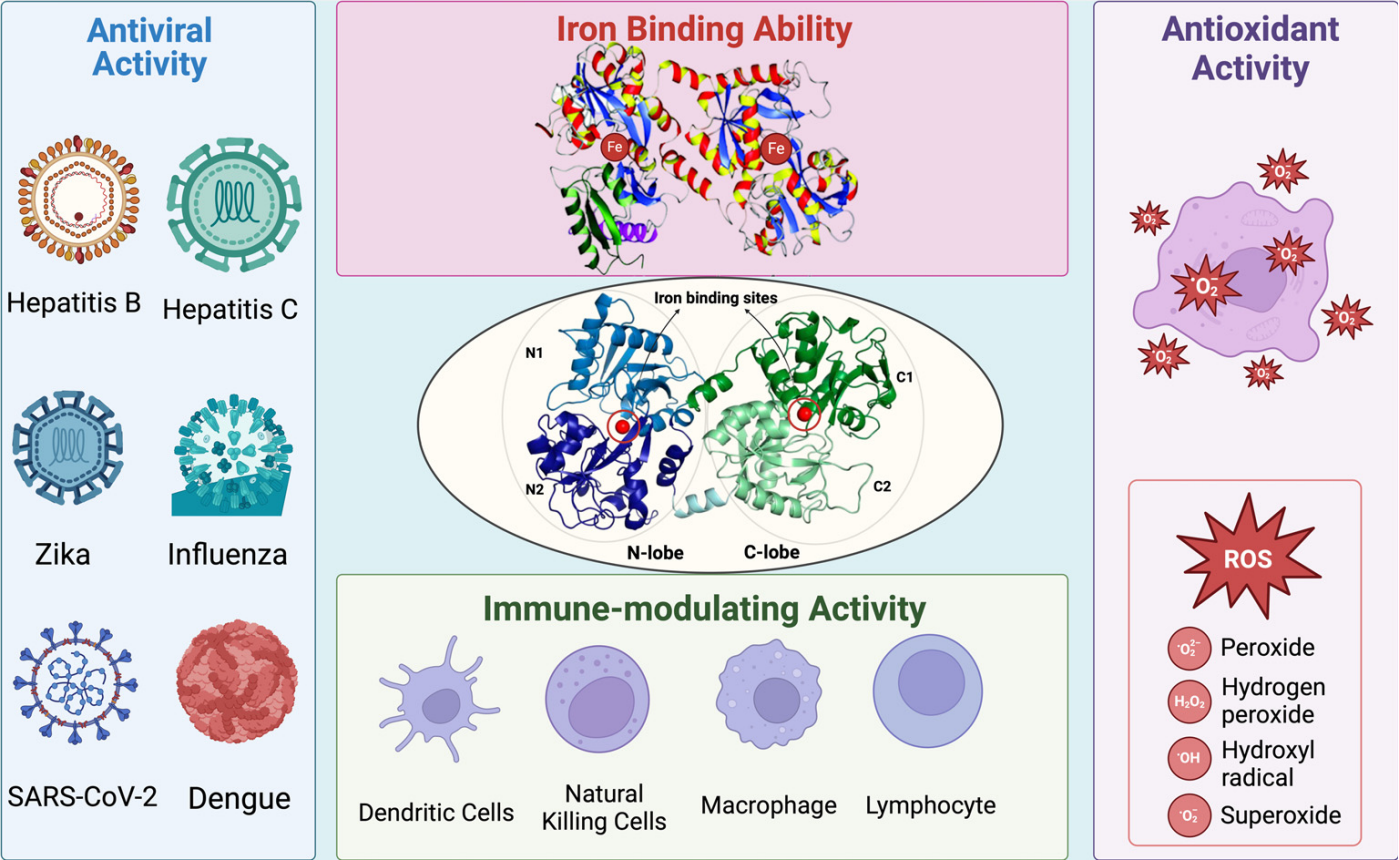
Summary of general structure and biological activity of Lf. Lf possesses two lobes called N-lobe and C-lobe. Each lobe is equipped with an iron-binding site, contributing to its high affinity for iron ions. The iron-binding ability of Lf also enhances its antioxidant activity by preventing the Fenton reaction and thus the formation of ROS. Moreover, Lf exhibits significant antiviral activity against several type of viruses, including but not limited to hepatitis, influenza, Zika, coronavirus, and dengue. Since Lf is one of the main components of the immune system, it has a close relationship with other types of immune cells. Lf can interact with other main components of the immune system and induce an immune response against viral infections.

The antiviral activity of Lf has been extensively studied for a long time (9, 10), against both DNA viruses, such as herpes virus (11, 12) and adenovirus (13), and RNA viruses, such as Japanese encephalitis virus (14) and echovirus (15). The primary mechanism of its antiviral activity involves the direct interaction of Lf with viral proteins and host cell surface molecules that are targeted by viruses, thereby interfering with viral infection. Furthermore, Lf can be released to exert antimicrobial, anti-inflammatory, and immunomodulatory effects by regulating the immune response (e.g. by promoting natural killer cells) and controlling acute and chronic inflammation (e.g. by regulating cytokine levels) (16–18) (**Figure 1**).

The properties of Lf have been utilized for both prevention and treatment of various diseases (19), such as COVID-19 (10) and chronic hepatitis C (20). In this review, the known and potential mechanisms behind the antiviral activity of Lf are discussed and summarized. The range of viruses for which Lf can serve as an antiviral agent is narrowed down to specific types with significant potential to cause viral infectious diseases worldwide. In addition, the immunomodulatory activity of Lf in certain viral infections is briefly summarized to provide an overview of how Lf interferes with host-virus interaction and modulates the immune response.

## 2 General structure and structure-related biological activity of Lf

Lf consists of two homologous, spherical, and symmetrical lobes called the N-lobe and C-lobe. Each lobe contains two domains and can bind a single iron atom, with the lobes connected by a short alpha helix (3, 4). The surface of Lf is positively charged, which enables it to bind with anionic molecules found on certain pathogenic microorganisms and host cell surface components. These interactions form the basis for most of Lf’s antipathogenic activities, preventing the connection between host and pathogen and interfering with target receptor binding.

The efficiency of Lf’s activities may vary depending on whether it is in an iron-bound (Holo-Lf) or iron-free form (Apo-Lf). The conformation of the N-lobe changes from an open to a closed structure upon iron binding (21). This conformational change affects the resistance of Lf to proteolysis, as the structural state becomes more stable with iron binding. When both lobes of Lf are covalently bound to iron, the molecule becomes more stable and compact, making it resistant to digestion and iron deprivation (22).

A beneficial aspect of Lf’s iron-binding ability is its high affinity for iron, which enhances its use in the treatment of iron-related disorders and diseases at both low and high levels of iron saturation. A similar finding was reported in an *in-vitro* study on the neuroprotection activity of Lf (23). Both the Holo-Lf and Apo-Lf were tested for their potential neuroprotective effects on cultured ventral mesencephalon neurons exposed to oxidative stress. Holo-Lf demonstrated a neuroprotective effect by maintaining oxidative stress levels without releasing its bound iron ions into the environment.

In a review of the structure of Lf (22), it was noted that domain motions are the primary variable regulating the transformation between the Apo-Lf and Holo-Lf. Despite changes in the stabilization and rigidity of the molecule, no significant alterations are observed in the surface region. This suggests that certain activities of Lf, such as antibacterial and antiviral functions, remain intact since the receptor interactions are not affected. These structural properties of Lf contribute to its multifunctional features and stability.

The antibacterial activity of Lf against Gram-negative bacteria is primarily mediated by its positively charged N-terminus, which interacts with the lipopolysaccharide on the bacterial cell surface (24). This interaction disrupts the binding of lipopolysaccharides to specific cations, resulting in bacterial cell damage. A similar activity is also observed against Gram-positive bacteria, where the positively charged Lf binds to anionic structures on the bacterial cell surface, enhancing enzymatic activity that leads to cell lysis (24).

The significance of the N-terminus of Lf in antiviral activity is further demonstrated by Lf-derived peptides. Lactoferricins are bioactive natural antimicrobial peptides that are produced in digestion, comprising the N-terminal region of the Lf (25). In addition to their antiviral properties, lactoferricins are known with their potent antimicrobial activities (antifungal, antibacterial, and antiparasitic), along with anti-inflammatory and immunomodulatory properties (26). As highlighted in this review, the N-terminal region of Lf is crucial for the protein’s antiviral activity, largely due to its positive charge.

Lactoferricins retain most of the essential characteristics of Lf and can exhibit stronger activity than Lf itself in terms of efficiency (26). The positive charge and positions of cationic residues are crucial factors contributing to the enhanced activity of lactoferricins (27). These factors are so significant that they affect the efficiency of peptides derived from different Lf sources. Among these, lactoferricins derived from bovine Lf (bLf) typically show stronger activities, particularly in antimicrobial studies, compared to other commonly used lactoferricins (27). Such distinctions should be carefully considered in lactoferricin-based antiviral studies.

As previously mentioned, the iron-free form of Lf exhibits higher antibacterial activity, due to its ability to remove essential iron ions for the growth of pathogenic bacteria (21). One distinguishing factor between Lf and transferrin is Lf’s high affinity for iron, even at low pH values. While transferrin cannot bind iron at a pH of ~5.5, Lf retains its binding capacity at a pH as low as ~3.5 (28).

Additionally, these properties contribute to the antioxidant activity of Lf, which is currently being investigated in the context of iron accumulation and the treatment of reactive oxygen species (ROS). The antioxidant activity of Lf is also influenced by its level of iron saturation. Similar to its antibacterial activity, the antioxidant activity of Lf are enhanced by its extensive interaction with iron ions. If enzymatic protection is insufficient or absent, iron ions can react with peroxides in a Fenton reaction, forming highly reactive hydroxyl radicals that cause cellular damage through oxidative stress (29).

Studies have demonstrated that Lf’s scavenging activity against hydroxyl radicals produced by the Fenton reaction prevents oxidative DNA damage *in vitro* (30). The antioxidant activity of Lf is widespread throughout the human body, reducing oxidative stress in various cell types. For instance, research has shown that bLf exerts an antioxidant effect on intestinal Caco-2 cells and liver cell lines (31). Furthermore, Apo-Lf can also exert antioxidant effects on dopaminergic cells by reducing the rate of Fenton reaction in the brain (22).

## 3 The antiviral mechanism and the triggering of the host immune response by Lf

### 3.1 Lf interferes with the cellular attachment of viral particles

**Figure 2:**
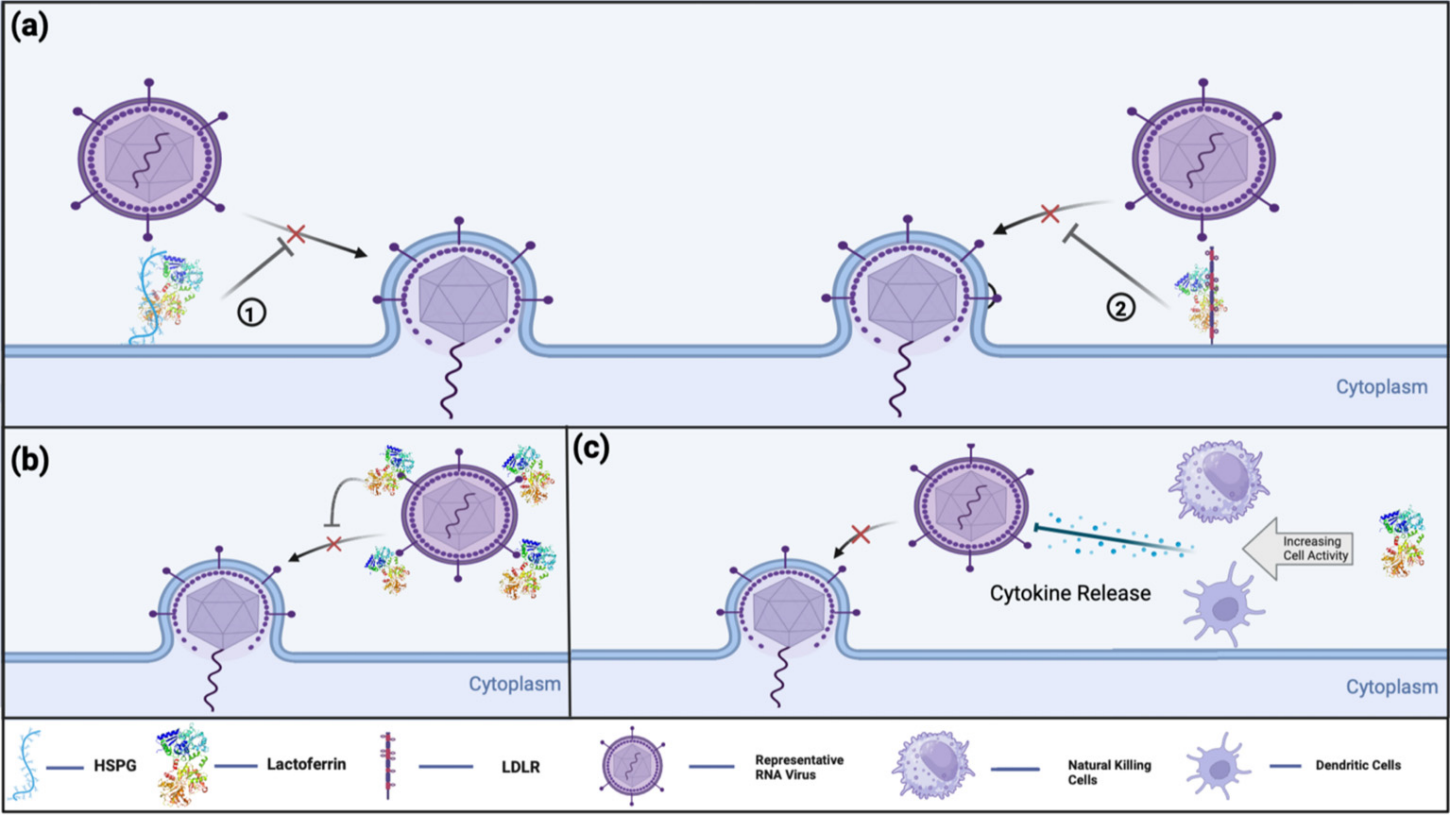
Current known antiviral mechanisms of Lf against viral infections include: **(A)** Lf’s receptor compatibility against viral infection. (1) Lf binds to HSPG on host cell receptors, preventing virus-host cell interaction. (2) Lf interferes with virus-host cell interaction by binding into LDLR, thereby preventing or decreasing the efficiency of viral infection **(B)** Lf’s direct antiviral activity by virus binding. Lf binds directly to virus-targeted receptors to prevent the initiation of virus-host cell binding. **(C)** Lf’s indirect antiviral activity by immune modulation. Lf promotes immune cells to modulate cytokine release and enhance the host immune response against viral infections.

The cationic structure of Lf is a highlighted feature that facilitates its interaction with negatively charged viral surface proteins, such as DNA molecules and glycosaminoglycans (GAGs) (17). The general mechanisms of Lf’s antiviral activity can be summarized as shown in the **Figure 2** (21):

- Binding to host cell surface molecules to interfere with virus attachment and entry: Heparan sulfate proteoglycans (HSPGs) are the best-known and most-studied surface molecules associated with Lf. The disruption of viral binding for certain viruses is observed through the interaction between Lf and HSPGs, but is not limited to these molecules. Lf can also exhibit similar activity with other important surface molecules, such as GAGs and low-density lipoprotein receptors (LDLR), which are discussed in the later parts of this review.
- Direct binding to viral particles and receptors, leading to inhibition of viral adsorption: Lf can interfere with the interaction between the virus and the host cell in multiple ways, as it can bind to the virus, the host cell, or sometimes both, depending on the administered concentration.
- Indirect antiviral activity through immune modulation by Lf: These intracellular activities of Lf can be further extended, as suggested by a study indicating the potential inhibition of viral enzymes (32).

These molecular mechanisms of Lf can vary depending on the type of virus (33). Additionally, the type and structure of Lf can influence these molecular mechanisms. For example, certain forms of Lf have demonstrated higher antiviral activity against the same type of virus when compared to others. Likewise, the efficiency of Lf’s activity can be affected by whether it is in Apo-Lf form and Holo-Lf form. **Table 1** illustrates the variations in mechanisms of action different types of viruses.

**Table 1:** Antiviral activity of Lf.

Type of Virus	Study Model	Mechanism Of Action	Results That Indicate Antiviral Activity of Lf	Reference
SARS-CoV-2	*In-vitro*	Binding into receptor binding domain of S protein	Reduction in SARS-CoV-2 titers in Vero cells by Apo-Lf and Holo-Lf	(34)
SARS-CoV-2	*In-vivo* *In-vitro*	Prevention of post-infection of SARS-CoV-2 by Lf-Zn-NPs with possible enzymatic suppression Interference of Spike RBD and ACE2 Potential virucidal activity	*In-vitro* antiviral activity through ACE2 receptor binding Decrease in the binding affinity between RBD and ACE2 (enhanced with Zn-NPs) Lung biomarker levels in a rat model significantly increased by Lf-Zn-NPs administration to control levels	(35)
SARS-CoV-2	*In-vitro* *In-vivo*	Disruption on viral replication and assembly in post-infection stage Potential suppressing activity Potential inhibition of RNA-dependent RNA polymerase activity Interaction with RBD of the spike protein	Decrease in the nucleocapsid protein production of SARS-CoV-2-related coronavirus GX_P2V Successful prevention of viral entrance in different variants, ranging from 72% to 93% Partial inhibition of RNA polymerase activity Suppression of viral copes in lung and trachea regions in an *in-vivo* hamster model	(36)
SARS-CoV-2	*In-vitro*	Disruption of cathepsin-assisted cell entry pathway in certain variants	Suppression of viral entry in Wuhan pseudoviruses and Omicron variants in Vero cells Increased antiviral activity by inhibition of TMPRSS2 activity, possibly by shifting the virus’s interaction to the ACE2 receptor	(37)
SARS-CoV-2	*In-vitro*	Inhibition of early stages of infection by preventing viral entry	The difference between the Apo- and Holo- form of bLf was not significant on reduction in SARS-CoV-2 infection (71% and 74% in ancestral, 65% and 67% in omicron strain, respectively). In terms of prevention of viral entry, holo-bLf showed higher activity than the apo-bLf.	(38)
Dengue	*In-vitro In-vivo*	Binding into the cellular membrane of the virus Potential interaction with HS and DC-SIGN receptor Compatibility between bLf and LDLR that causes prevention of virus’s interaction with the host cell.	bLf treatment at 25 μg/mL and higher concentrations significantly decreases the infection rate of DENV in Vero cells bLf treatment decreases morbidity by 60% on DENV-infected mice	(39)
Zika and Chikungnya	*In-vitro*	Possible interaction with host cell receptors such as HSPG	Successful prevention of Chikungunya virus and ZIKV infection in Vero cells by nearly 80% Influenced antiviral activity of Lf depending on the administration stage in both viruses	(40)
Influenza	*In-vitro*	Binding into viral proteins of several Influenza strains Competitive substrate potential of bLf -bound sialylated glycans by preventing attachment of Influenza to host cells	Significant decrease (30% to 60%) in viral protein interaction after the hydrolyzation of sialylated glycans	(41)
Influenza	*In-vitro*	Direct binding to the virus on uncoating stage of infection Potential enhancement of surface molecule interaction by the conformational change of hemagglutinin	Viral antigen synthesis was decreased by 70% Infection were prevented by 90% No significant antiviral activity after the first hour pH-dependent viral binding was observed, along with most efficient rate in pH 5 The C-lobe of the protein significantly influences the antiviral activity	(42)
Influenza	*In-vitro*	Inhibition of Cascape 3 activity Prevention of viral ribonucleoprotein exportation	Apoptosis rate of infected Madin-Darby canine kidney (MDCK) cells decreased by ~85% Potential effect of Lf as trapping the viral ribonucleoproteins inside the nucleus of the virus (influenced by decreased Cascape 3 activity)	(43)
Influenza	*In-vitro*	Prevention of hemagglutinin action by C-lobe of Lf	Significant viral replication decreases by virus binding inhibition	(44)
Influenza	*In-vitro*	Virus attachment disruption derived by cell receptor competition Potentially prevention of viral ribonucleoproteins exportation	Inhibition of influenza infection in Madin-Darby canine kidney epithelial cells The desialylated and deglycosylated forms of Lf positively alter the inhibition rate Cytopathic effects were significantly decreased during Lf administration in adsorption and the whole step process Unrestricted antiviral activity in terms of ion-binding, glycosylation, or sialylation	(45)
Hepatitis C	*In-vitro*	Negative influence on the intracellular activity by possible inhibition of viral Helicase/ATPase	HCV replication was significantly decreased on Huh-7 cells Possible intracellular activity by inhibition of viral Helicase/ATPasemean The intracellular activity does not include viral RNA polymerase.	(32)
Hepatitis C	*In-vitro*	Intracellular replication and cellular entry of HCV were negatively influenced by Camel Lf and its N/C lobes	Viral entry of HCV was inhibited in Huh 7.5 cells Changeable antiviral activity based on the dose and type of agent	(46)
Hepatitis C	*In-vitro*	Inhibition of HCV viral replication by different types of Lf	Type-based antiviral activity against HCV in HepG2 cells in terms of efficiency Successful prevention of viral entry in all types, dose-dependently Observable intracellular activity during HCV infection, influenced by the Lf type	(47)
Hepatitis B	*In-vitro*	Successful binding into HBV surface antigen Inhibition of HBV infectivity	Significant antiviral activity by inhibition of viral binding in HepG2-NTCP cells in all types of Lfs Dose-dependent reduction of HBV infectivity by a decrease in antigen levels and HBV DNAs	(48)
Hepatitis B	*In-vitro*	Disruption of the interaction between the viral particles and host cells	Significant inhibition of HBV infection by hLf-GAG interaction	(49)
Hepatitis B	*In-vitro*	Significant inhibition of HBV DNA by bLf	Holo-bLf exhibited antiviral activity against HBV, meanwhile Apo-bLf did not Structural change by the iron binding can influence the antiviral activity of bLf	(50)

GAGs are negatively charged long polysaccharides that can be classified as either sulfated, such as heparin or HSPGs, or non-sulfated, such as hyaluronic acid (51). Most viruses initiate infection by binding to glycans or carbohydrate-based structures on the cellular surface. As a type of glycans on the cell surface, GAGs are one of the most important targets during the early stages of viral infection. HSPGs are a specific type of GAG that contain at least one covalently linked heparan sulfate (HS) chain (52). HSPGs are mainly found on the cell surface and, to a lesser extent, in the extracellular matrix, where they cooperate with adhesion receptors to facilitate cell-cell interactions.

Sulfated GAGs, especially HS, carry a high negative charge (33). For this reason, HSPGs are frequently targeted by viruses as the primary site for initiating host-cell contact (53). The negative charge of HSPGs allows them to interact electrostatically with viral surface glycoproteins and capsid proteins, thereby they are naturally targeted by viruses (54). The cationic structure of Lf enables it bind with HSPGs, disrupting the virus’s preferred entry route into the host cells (53).

Moreover, the interaction between Lf and HSPG is not limited to antiviral studies. For instance, in dopaminergic cells, Lf may regulate intracellular signaling pathways to increase cell survival during Parkinson’s disease pathogenesis, potentially interacting with HSPGs. However, the current understanding of the neuroprotective activity of Lf, and the role of HSPGs in this interaction, remains incomplete. It is suggested that Lf may promote intracellular activity that enhances cell survival by interacting with HSPGs (55). Therefore, Lf may protect certain cell types in different parts of the human body from toxicity or viral infections. This article partially discusses the potential intracellular activity of Lf based on its antiviral properties. Future studies may reveal that the antiviral activity of Lf, related with HSPGs, is not only derived from receptor competition with viral particles but also involves chain reactions that enhance cellular survival and/or host cell resistance against infection.

LDLRs are glycoproteins that regulate blood cholesterol levels by mediating the endocytosis of cholesterol-containing lipoprotein particles from the bloodstream (39, 56). These cell surface molecules are also potential targets for viral entry, as certain viruses use them to infect host cells. Lf has been shown to bind to the LDLRs in some cells, thereby influencing specific cellular pathways. For example, Lf can promote the proliferation of osteoblasts by affecting the ERK1/2 signaling pathway through its binding to LDLRs (21). A similar intracellular effect of Lf was observed in another study, where Lf was found to increase tropoelatin levels by activating lipoprotein receptor-related protein-1 via the PI3K/Akt signaling pathway (57). While these studies are beyond the scope of this review, they are mentioned to highlight the cellular level activity of Lf in its interactions with LDLR and lipoproteins.

The interaction between Lf and LDLRs has also been discussed in the context of antiviral activity. In one study, the relationship between LDLR, dengue virus (DENV),and bLf was examined (58). The results indicated that LDLR might facilitate DENV entry into the cells, and bLf can inhibit the infection rate by binding to LDLR. Although the role of LDLRs in viral infection cycle appears to be less significant than HSPGs (as covered earlier in this review), future findings on LDLRs may provide valuable insights into the antiviral activity of Lf and its impact on host-virus interactions.

Another study investigated the role of LDLRs in the life cycle of HCV (39). Treatment with LDLR-specific antibodies significantly reduced viral RNA replication, suggesting a direct relationship between LDLRs and HCV. While these results indicate that LDLR is not an essential receptor for HCV, optimal replication levels were observed when LDLR presence and activity were mediated *in vitro*. Additionally, similar studies demonstrated that HCV stimulates LDLR expression in infected Huh7 cells, enhancing their interaction with LDLR (59).

These findings suggest the availability of surface proteins is a crucial factor in determining Lf’s antiviral efficiency. If a virus has alternative surface molecule to initiate entry, receptor blocking by Lf will be less significant. In contrast, if a virus relies specifically on a single type of surface molecule, there is a greater chance to observing significant antiviral activity with Lf treatment.

For instance, as discussed in subsequent sections, Zika virus (ZIKV) does not dependent on HSPGs for cellular entry. Consequently, Lf might be less effective against ZIKV when administered before the infection phase (as covered in the ZIKV section). The specific characteristics of a virus can significantly impact the antiviral activity of Lf.

Another example of this can be seen in Lf’s antiviral activity against SARS-CoV-2. Previous research has identified the interaction between the S protein of SARS-CoV-2 and GAGs (60). The antiviral activity of bLf may be mediated by binding to GAGs, creating competition with the virus, or by directly binding to the viral particles, as observed in different viruses, including SARS-CoV-2 (61).

Furthermore, Lf’s antiviral activity against hepatitis virus has also been demonstrated (47, 49). Studies indicate that, Lf can inhibit the entry of hepatitis B virus (HBV) and hepatitis C virus (HCV) through competitive binding to GAGs and direct interaction with viral particles, respectively. As shown in **Table 1**, Lf exhibits additional mechanisms against HCV and HBV, such as possible intracellular influences and surface antigen interaction. For HCV, Lf’s interaction is also mediated by binding to the envelope proteins E1 and E2 (62). The E2 protein primarily mediates the interaction between HCV and GAGs (63). Therefore, by preventing the interaction between HSPGs and HCV, Lf exhibits multiple antiviral mechanisms. These findings demonstrate that Lf can show various antiviral mechanisms against different viruses.

### 3.2 Lf initiates host responses in viral infection by immune modulation and activation

Natural killer (NK) cells are known to trigger immune responses against viral infections (64). These cells play a crucial role in mediating cellular homeostasis and synthesizing cytokines to eliminate infected cells (65). Viral infection of the host cell induces changes that enable NK cells to recognize and target the infected cells. For instance, this recognition occurs in flavivirus infections, such as during DENV infection, and potentially in influenza (64). A review article highlighted the specific interaction between NK cells with DENV, demonstrating that NK cells can engage with DENV through multiple specific receptors during infection (66). This interaction involves a complex network of surface receptors, where NK cells are activated by recognizing viral peptides and soluble factors produced by infected cells. Additionally, the frequency of some of these receptors increases during the DENV infection, thereby strengthening the impact of NK cells on the infection from a genetical perspective.

The immunomodulatory activity of Lf stimulates responses in certain immune cells, such as lymphocytes and NKs (16). These interactions enhance activities, such as cytokine production, proliferation, and migration. Thus, Lf can modulate immune responses by exhibiting both pro-inflammatory and anti-inflammatory effects in certain viral infections (67).

A study related to the topic of the article explores the effect of orally administered bLf on different mouse models with hepatitis (68). The results suggest that bLf administration offers protection against hepatitis and increases the expression of IL-11 in the small intestine of mice, indicating that Lf can regulate the immune response during the intestinal infection. Increased IL-11 expression can decrease the production of inflammatory cytokines. Given Lf’s significant impact in the intestinal regions, it may also provide protection near infected areas, along with a potential antiviral activity. Another study investigated the response of neonatal mice vaccinated against influenza A to treatment with Lf (69). The administration of Lf significantly increased the production of anti-influenza A antibodies and IgG against the infection. The researchers indicated that recruitment and activation of dendritic cells are behind this effect. However, Lf seems to positively influence immune cell activity by interacting with dendritic cells rather controlling a response from the system. These different outcomes suggest that Lf selectively influences the immune cells for the benefit the host.

Additionally, a randomized double-blind study demonstrated the prevention of symptoms and regulation of host inflammatory responses by a combined treatment of bLf and IgF against the common cold (70). IgF, an Ig-rich fraction of whey protein, has a crucial role in the immune system. As expected, the combined treatment significantly reduced the inflammatory response, decreased the number of symptoms, and enhanced the immune response. This cooperation between Lf and other immune system compounds indicates its multidirectional interaction. Finally, the immunomodulatory effect of Lf was recently investigated in coronavirus disease 2019 (COVID-19). Pretreatment with Lf against SARS-CoV-2 may be able to control cytokine expression, potentially mitigating the cytokine storm triggered by SARS-CoV-2 (71). Similar to the earlier study, Lf can effectively control the overproduction of inflammatory cytokines during infection.

Although not directly related to antiviral effects, studies investigating the immune modulation and anti-inflammation properties of Lf-derived peptides have been conducted. For example, the control of intestinal inflammation by bLf derived peptides, lactoferricin-lactoferrampin, was demonstrated in a study (72). The effect of these two peptides against intestinal inflammation was observed *in vivo* in piglets. Blood tests showed the following results: a significant reduction in total white blood cell and lymphocyte count to control levels, a significant increase in IgM and IgG antibodies (exceeding control levels), and a significant reduction in pro-inflammatory cytokines to control levels. Another Lf-derived peptide, LFP-20, derived from porcine, demonstrated promising results in modulating inflammatory response and immune homeostasis in multiple studies by the same researchers (73, 74). Results from both of these studies showed that LFP-20 significantly controls the cytokine levels (such as various ILs and TNF-α) and upregulates the secretion of diverse Igs (such as IgE, IgG, and IgA). Although the range of these studies is limited, it is clear that Lf has significant potential as an efficient antiviral agent by enhancing the host immune response against various diseases. Moreover, considering its immune regulation activity, Lf-derived peptides exhibit high potential in controlling virus-induced immune responses and initiating host response under specific conditions. Combined treatment with both Lf and Lf-derived peptides could significantly enhance antiviral strategies. A combination of these two characteristics could advance current antiviral research, both in context of infectious diseases and from the Lf perspective.

## 4 Antiviral activity of Lf against different virus types

In recent years, at least ten major epidemic viruses have emerged, including coronaviruses, flaviviruses, togaviruses, filoviruses, and orthomyxoviruses (75). Some of these viruses are capable of causing infectious diseases that pose significant threats to global health security. This review focuses on several of these viruses that fall into this category: Severe acute and Middle East respiratory syndrome, ZIKV, Chikungunya, Viral hepatitis, DENV, Ebola, and Pandemic influenza (76). Global changes are rapidly altering key aspects of infectious diseases, such as their dynamics, modes of transmission between populations, and risk of emergence (77). Factors like climate change and technological improvements can shift the geographical range of the disease vectors and create new interactions between species, potentially leading to zoonotic outbreaks like SARS-CoV-2. While global advancements have generally increased healthcare capacity and reduced the risk of infection for many viruses, there remains a need for novel and improved agents in viral infection research.

Much of the current research on viral infectious diseases focuses on either enhancing existing antiviral molecules or discovering new alternatives, particularly those with therapeutic potential. In this context, Lf’s antiviral activity aligns well with ongoing research in antiviral studies. Lf has demonstrated *in-vitro* antiviral activity against various viruses. This review highlights the potential of Lf as an antiviral agent against viral infectious diseases, specifically focusing on, SARS-CoV-2, ZIKV, DENV, and hepatitis viruses. Additionally, this review discussed mechanisms underlying Lf’s antiviral activity, gaps in the existing literature, and the current state of the Lf research.

### 4.1 SARS-CoV-2

Coronaviruses are pathogenic enveloped viruses belonging to the *Coronaviridae* family, which includes four genera: α-, β-, γ- and δ- (78). SARS-CoV-2 is a β-coronavirus with a large, positive single-stranded RNA genome of approximately 30kb in length (79). SARS-CoV-2 interacts with host cells through its spike (S) protein, which recognizes the angiotensin-converting enzyme 2 (ACE2) membrane protein. The World Health Organization (WHO) declared COVID-19 pandemic in March 2020, just a few months after the first reported case (80). The rapid spread of COVID-19 worldwide promoted extensive research on SARS-CoV-2 across various scientific fields. These efforts aimed to enhance understanding of coronaviruses and SARS-CoV-2, particularly to develop vaccines and identify potential molecular agents for treatment and post-treatment to alleviate symptoms.

Lf was one of the antiviral agents tested in various SARS-CoV-2 research and showed promising results. In a particular study, both bLf and hLf were evaluated as antiviral agents against SARS-CoV-2 spike-decorated pseudoviruses in certain cell types (81). The study also demonstrated that bLf could prevent increases in ferritin and proinflammatory levels. Both bLf and hLf exhibited significant inhibitory activity against Vero E6 cells treated with spike pseudoviruses, with bLf showing slightly higher activity. Similarly, in a human bronchial epithelial cell line (16HBE14o) infected with SARS-CoV-2, Lf displayed comparable antiviral effects, although with slightly less activity than in Vero E6 cells. Additionally, the study showed for the first time that the interaction of bLf with the S protein depends on the oligomerization state of the Lf. Furthermore, bLf exhibited a protective effect against iron overload and inflammatory disorders induced by the S protein on Caco-2 cells.

An interesting study on the interaction between Lf and SARS-CoV-2 was based on the hypothesis of molecular mimicry of the receptor binding domain (RBD) of the SARS-CoV-2 spike protein with transferrin (Tf) and Lf (34). The research jointly investigated the antiviral activity of Tf and LF, focusing on antibody cross-reactivity and competition against spike proteins. Discontinuous electrophoresis results indicated that the interaction between RBD and Lf was more significant compared to the interaction with the spike protein itself. The competition between RBD and Lf in binding to the transferrin receptor was observed, which also contributed to reducing the infection rate. These findings were reflected in titration results, showing significantly higher activity against the Wuhan and Delta variants on Vero cells treated with Holo-Lf and Apo-Lf.

Additionally, it has been reported that Lf targets specific pathways and complexes to inhibit SARS-CoV-2 infection. For instance, bLf and milk fat globule membranes were tested against SARS-CoV-1 and 2 infections in both *in vitro* Vero cells and *in-vivo* hamster models (36). Both molecules effectively reduced the production of nucleocapsid protein of the SARS-CoV-2-related coronavirus GX_P2V to nearly zero without causing toxicity. Similar positive results were observed in different human cell line infections with the same type of coronavirus. Afterwards, bLf’s activity at the viral entry stage of SARS-CoV-2 was evaluated. At a specific concentration, bLf successfully prevented the entry of different viral variants, with an efficiency ranging from 72% to 93%. The affinity of bLf for the SARS-CoV-2 spike protein was also examined, and its interaction with the RBD of S protein was identified at the N-terminus of bLf in the docking model of the experiment. The study also addressed RNA-dependent RNA polymerase (RdRp) activity of both SARS-CoV-1 and 2, given their high similarity, with bLf treatment. The antiviral activity of the bLf in post-entry stage was demonstrated by the inhibition of RdRp *in vitro*. The results revealed that the dose-dependent antiviral activity of bLf was mediated through its interaction with nsp12, the catalytic subunit of the RdRp. Several potential hydrogen bonds corresponding to this interaction were identified during the experiment. Given the significant similarity between the RdRp of SARS-CoV-1 and SARS-CoV-2, it can be assumed that bLf exhibits strong antiviral activity through RdRp inhibition in both viruses. In the final part of the study, an *in-vivo* hamster model demonstrated that bLf treatment significantly suppressed viral copies in the lung and trachea regions.

A similar study identified Lf as a key agent behind the antiviral activity of human breast milk against SARS-CoV-2 (82). However, this experiment found that multiple proteins, including Lf, mucin 1 and α-lactalbumin, contributed to SARS-CoV-2 inhibition. Infected Vero E6 cells were treated with a mixture of Lf, mucin 1 and 4, lactadherin and α-lactalbumin to determine which compounds were effective antiviral agents in addition to Lf. While mucin 4 and lactadherin showed no activity against infected cells, mucin 1 and α-lactalbumin at a concentration of 0.25 mg/mL exhibited significant inhibition of SARS-CoV-2 pseudovirus infection, achieving an inhibition rate of more than 60%. Moreover, the effect of these compounds on the expression of a reporter gene and viral RNA were also examined. For Lf, multiple types-recombinant, human, goat and bovine-were tested, and all demonstrated downregulation of both reporter gene expression and RNA levels. Similar results were observed for mucin 1 and α-lactalbumin, although α-lactalbumin exhibited relatively lower impact. Investigating the mechanisms behind the inhibition of SARS-CoV-2 infection revealed that all proteins exhibit antiviral activity during phases of viral attachment and entry, while only Lf and mucin 1 maintained their activity after the viral replication phase. Finally, α-lactalbumin was shown to interfere with the interaction between the ACE2 receptor and the S protein, while Lf and mucin 1 inhibited the attachment of the virus to HSPGs.

A study demonstrated the antiviral activity of Lf against SARS-CoV-2 infection by interfering with the S protein-ACE2 interaction through its binding to the co-receptor HSPG (5). Infected cell cultures were treated with bLf at various phases of infection. The results indicated that bLf exhibited antiviral activity by inhibiting viral replication through the prevention of virus attachment. The attachment experiments showed that bLf binds to HSPG on the cellular surface, thereby disrupting the HSPG-mediated S protein-ACE2 binding, and preventing viral interaction. The binding of Lf to HSPG was confirmed by a reduction in antiviral activity in the presence of heparin and further supported by docking models.

Similar study demonstrated that Lf interferes with the interaction between the spike protein and HS interaction in SARS-CoV-2 infection (83). To investigate the receptor interaction of SARS-CoV-2 in absence of HS, Lf was used during the experiments. The results showed that Lf successfully reduced the attachment of the virus’s receptor-binding domain to HS.

Another *in-vitro* experiment on Vero cells found that bLf inhibits the entry of SARS-CoV-2 Wuhan pseudoviruses into Vero cells by targeting the ACE2 receptor (37). The Wuhan strain of SARS-CoV-2 relies on transmembrane protease serine 2 (TMPRSS2) to infect host cell. The inhibition of TMPRSS2 activity increased the antiviral activity of bLf by preventing the TMPRSS2-mediated interaction between the ACE2 receptor and the virus. Based on the experiment, these findings suggest that bLf has the potential to interfere with the cathepsin-assisted entry of the virus. The study also highlighted the effective inhibition of Omicron variants with bLf treatment, possibly by interfering cathepsin-mediated infection. The final experiment in the study showed that bLf suppresses the activity of mRNA coding interferons, which are induced by SARS-CoV-2 during the first 24 hours of infection.

Given their well-known properties, Lf peptides, including lactoferricins, have been extensively investigated against SARS-CoV-2 infection.

One particular study demonstrated the potential inhibition activity of lactoferricins (peptide GSRY) against the SARS-CoV-2 main protease (Mpro) (84). The Lf-derived peptide GSRY was tested against Mpro and compared with inhibitor N3 (the main ligand) in terms of binding affinity. The results revealed that binding of GSRY-Mpro complex demonstrated higher binding energy than the N3-Mpro complex, indicating a stronger binding strength of GSRY compared to the inhibitor. The analysis of the molecular interaction between GSRY and Mpro showed that multiple hydrogen bonds and carbon-hydrogen bonds mediated this interaction, along with one pi-alkyl and one charge interaction. The authors suggested that these results could support the use of Lf-derived peptides as a novel SARS-CoV-2 inhibitors.

Moreover, a recent study showed a direct interaction between lactoferricins and the receptor-binding domain of the SARS-CoV-2 S protein (85). The first experiment demonstrated the dose-dependent S protein binding by both bLf and hLf, predominantly at the ACE2 receptor. Then, Lf peptides from different regions, such as the N- and C-terminals, were tested with the S protein *in vitro*. It was found that, although Lf was the main inhibitor, the N-terminal peptide of Lf significantly reduced the binding capability of the S protein, whereas the C-terminal peptide demonstrated only partial, non-significant inhibition against the S protein. These results suggest that Lf primarily initiates S protein binding with its N-terminal region. In conclusion, the analysis of the S protein and Lf interaction shows that Lf not only blocks the interaction between SARS-CoV-2 and HSPG, but also inhibits the binding on primary receptor ACE2 during infection.

Another study investigating the inhibitory activity of Lf-derived peptides against SARS-CoV-2 focused on the role of TMPRSS2. The study explored how inhibition of TMPRSS2 by Lf and synthetic Lf-derived peptides could prevent SARS-CoV-2 infection *in vitro* (86). A peptide derived from the N-terminal region of Lf showed the most significant inhibition of TMPRSS2 proteolytic activity, Thereby preventing the proteolytic processing of the SARS-CoV-2 S protein, which is necessary for viral entry. While full-length Lf did not directly inhibit TMPRSS2 activity, it efficiently inhibited SARS-CoV-2 through several independent mechanisms. The Lf-derived peptide, however, demonstrated nearly 45% inhibition of TMPRSS2. In the final experiment, lactoferricins were also tested for their inhibitory effect against TMPRSS2. The results confirmed that lactoferricins could inhibit SARS-CoV-2 infection in Vero cells.

Besides its antiviral activity, Lf is also considered a potential therapeutic and dietary supplement for COVID-19 due to its iron-balancing properties and its ability to enhance and regulate the immune system (87). A clinical trial (88) highlighted the therapeutic potential of Lf by administering liposomal bLf to COVID-19 patients. In the study, 92 patients were divided into three groups: 32 received bLf, 32 received only the standard of care regimen, and 28 were untreated COVID-19 patients. The groups treated with bLf showed a significantly lower number of symptoms and a notable reduction in IL-6, ferritin, and D-dimer levels (88).

The global pandemic triggered by SARS-CoV-2 has caused a worldwide impact across many sectors, with some areas still experiencing its negative effects. This extensive impact makes SARS-CoV-2 one of the most important topic in pathology research. Investigations into the antiviral activity of Lf have been at the forefront of recent studies due to data suggesting its promising potential against SARS-CoV-2 and COVID-19. Future studies exploring the relationship between SARS-Cov-2 and Lf should be specifically designed to generate unique data that can advance research in both fields.

### 4.2 Zika virus

Mosquito-borne flaviviruses, transmitted by certain mosquito species, pose a global threat by infecting over 400 million people annually (89). These viruses are single-stranded, positive-sense, enveloped RNA viruses that include DENV, ZIKV, as well as Saint Louis encephalitis virus, West Nile virus, Murray Valley encephalitis virus, and Japanese encephalitis virus, which are not covered in this review (90). Among these, ZIKV is notable for its ability to cross the placental barrier (91).

GAGs serve as common receptors facilitating the infection of flaviviruses (36). ZIKV, for instance, initiates viral entry by binding to HSPGs on the surface of the host cell through its E protein, along with interactions with additional receptors (92). Once inside the host cell, the viral genomic RNA is translated into a single polyprotein, which is then cleaved to produce viral proteins, undergoing a replication process similar to other RNA viruses. The involvement of GAGs in the entry process of flaviviruses underscores the potential significance of Lf in antiviral studies. Despite this potential, only a few studies specifically investigate the antiviral activity of Lf against flaviviruses, particularly ZIKV and DENV. Moreover, some studies suggest that while DENV relies on HPSG for cell entry, ZIKB may not depend on these glycoproteins (54).

To give a sole example, the antiviral activity of bLf against chikungunya virus and ZIKV was investigated using Vero cells derived from the kidney of an African green monkey (40). The study showed minimal cytotoxicity of bLf and demonstrated a dose-dependent antiviral activity that prevented infection by up to 80% at a concentration of 1.0 mg/ml. Notably, bLf exhibited higher antiviral activity when administered during the virus addition phase compared to post-virus exposure, and to a lesser extent when given before virus infection. This suggest that bLf may exert its antiviral effect by blocking virus-binding sites on the cell surface, such as HSPG. Furthermore, the study found that the infection rate of ZIKV was lowest when bLf was administered during the infection phase, suggesting potential intracellular activity of bLf, possibly targeting viral RNA or interfering with infection-related cellular pathways. Considering the controversial studies on the dependence of ZIKV on HSPG, the different activities of Lf, including potential intracellular actions, could play a role in ZIKV infection.

Flaviviruses which have emerged due to climate change, have spread to wide geographical regions, leading to increased infection cases (93). Although some studies suggest that ZIKV is not dependent on HSPGs for viral attachment, Lf administration may still impact its infection by disrupting these glycoproteins, with evidence pointing to potential intracellular activity of Lf in ZIKV infection. However, the current understanding of Lf’s intracellular activity is limited from an antiviral perspective, and further studies are required to clarify this role. Comprehensive research could provide new insights into the intracellular activity of Lf and its potential as an alternative therapeutic agent for ZIKV infection.

### 4.3 Dengue virus

DENV is a mosquito-borne, single-stranded RNA flavivirus that is considered the most dangerous among flaviviruses, causing approximately 20 million cases annually (91). Despite the virus’s dangerous potential, there are only two vaccines currently available in the field, Dengvaxia^®^ and Qdenga^®^.

The receptors involved in DENV entry can vary depending on the type of cell being infected (94). However, DENV has been shown to rely on HSPGs to initiate cell entry with its E protein in certain cell types (54). Consequently, as with other flaviviruses, DENV replication is significantly influenced by the presence of GAGs (95). The replication process of DENV is similar to that of other RNA viruses discussed in this article. After endosomal processing, the viral RNA is released for translation and replication in the endoplasmic reticulum, followed by protein assembly and maturation in the Golgi network, and finally, the mature virions are exocytosed (40).

Like ZIKV, DENV belongs to the *Flaviviridae* family, in which Lf has the potential to exhibit antiviral activity. Apart from the study discussed later in this section, no research directly examines the antiviral activity of Lf against DENV. However, some studies suggest a possible role for Lf in DENV infections. For example, a study in 2000 reported a 17.3% increase in Lf levels in the blood samples of DENV-infected children (96). Another study from 2016 also indicated a possible role of Lf in the anti-DENV immune response, based on increased serum Lf levels in DENV-infected patients (97). Since DENV relies on GAGs to initiate its cellular entry, Lf’s affinity for GAGs could play a significant role in DENV infection, making it relevant target in antiviral research.

One study demonstrated the inhibition of DENV infectivity by bLf, particularly through its interaction with HSPG (58). Similar to the previous study, Vero cells were infected with different DENV serotypes (DENV-1 to DENV-4) and treated with bLf at various stages of infection. The results indicated a dose-dependent antiviral activity of bLf against all four DENV genotypes, reducing the number of infected cells by 41%, 81%, 41% and 43%, respectively. The most effective reduction occurred when bLf was administered during the viral infection stage, followed by administration before viral attachment. The antiviral activity of bLf activity after infection and when administrated across all stages was significantly lower compared to other phases. DENV relies on HSPGs to bind to the target cells, and bLf exhibits significant antiviral activity when administered before DENV attaches to cells. Since Lf accumulates near HSPGs receptors, it effectively blocks DENV from initiating infection. Given its potential to interfere with early steps of a viral infection, bLf demonstrates strong antiviral activity. Although it may possess some intracellular activity to disrupt infection, a precise has not yet been identified. However, by occupying HSPG receptors, it could prevent the second cycle of the infection. Further experiments confirmed that the presence of HSPGs greatly affects the antiviral capacity of bLf. The study also suggested a role of LDLR in DENV infection, as evidenced by a 36% reduction in DENV infection after anti-LDLR antibody treatment. To explore these results, bLf was tested for its potential to interfere with the interaction between LDLR and DENV. Remarkably, bLf exhibited inhibitory activity by competing with cellular LDLR, achieving a 62% inhibition rate. Additional tests using HS-expressing and HS-deficient hamster ovary cells revealed that the infection rate decreased with increasing bLf concentration in HS-expressing cells. In contrast, the infection rates were significantly lower and unaffected by bLfs in HS-deficient cells, highlighting the role of HS in the antiviral activity of bLf against DENV.

The results indicate that the antiviral capacity of the bLf is heavily influenced by the presence of HS. This suggest that, while bLf may have potential intracellular effects, these responses might depend on HSPG interaction, showing the necessity of further investigation into the role of HSPGs in Lf activity. Finally, the antiviral activity of bLf was demonstrated in an *in vivo* mouse experiment, with two groups of 10 mice (with and without bLf) were tested. Four mice from the bLf-treated group become ill, while six remained healthy.

Compared to ZIKV, DENV shows greater promise for Lf’s antiviral research. The strong dependence of DENV on HSPGs for viral attachment makes Lf an excellent candidate to disrupt this interaction, supporting further research into this relationship. As discussed previously, the increasing global impact of flaviviruses and the limited data in the area create a significant need for further research efforts, similar to those for ZIKV.

### 4.4 Hepatitis

Hepatitis refers to a high degree of liver inflammation caused by a viral infection from hepatitis viruses (A-E) (98). It can present as either acute (lasting 6 months or less) or chronic (lasting more than 6 months) and may cause symptoms such as nausea, jaundice, and abdominal pain. Each hepatitis subclass differs, as the A, C, D and E types are an RNA viruses, while type B is a DNA virus. Lf has been studied mainly in relation to HCV, followed by HBV.

HBV is a DNA virus from the *Hepadnaviridae* family, transmitted through infectious blood and certain body fluids like semen and saliva (90). HBV can initiate viral entry by interacting with HSPG receptors (99). Since Lf exhibits great affinity for HSPG, it has a potential to interfere with the interaction between HBV and host cells, thereby disrupting cellular infection.

Breast milk is an important source of nutrition for newborns and contains bioactive components with antiviral and anti-inflammatory properties (100). Along with Lf, many other glycoconjugates in milk potentially exhibit antiviral and immune-regulating activities (101). These glycoconjugates can also be utilized by specific bacteria, such as *B.infantis*, which contribute to the growth of beneficial bacterial populations in infants (102, 103). Moreover, breast milk has a unique microbiome that promotes probiotic activity in newborns, transmitting beneficial microorganisms through lactation. However, there is also the possibility of transmitting non-beneficial microorganisms. Notably, when HBV is transmitted from mother to child during the neonatal period, the incidence of chronic infection is significantly higher than when infection occurs later in life.

A recent study demonstrated the inhibition of HBV infection by human breast milk and investigated HBV transmission through lactation (48). The study found that human whey binds effectively to HBV surface antigens (both serum and recombinant), achieving a nearly 90% inhibition rate, whereas bovine whey exhibited limited activity. Based on these significant results, the components responsible for the antigen binding activity of human whey were investigated, revealing that hLf is the major component binding to the HBV surface antigens. To confirm these findings, recombinant surface proteins and 3 types of Lf (recombinant hLf, hLf, and bLf) were tested, each demonstrating positive results with high inhibition efficiency. In cell culture experiments, both human whey and different types of Lf significantly inhibited HBV infectivity. Overall, the results suggest that Lf is the primary component of human breast milk responsible for its inhibitory effect on HBV.

HCV is an RNA virus from *Flaviviridae* family that comprises 7 major genotypes and 67 subtypes, with genotype 1 is responsible for about half of HCV cases (104). This genomic variability poses a significant challenge for vaccine development compared to other hepatitis viruses. Like HBV, the main source of HCV transmission is through infectious, contaminated blood. HCV infection can cause inflammation through metabolic pathways such as insulin resistance or oxidative stress, and can also induce cell necrosis through immune-mediated cytolysis. As an RNA virus, the replication cycle of HCV differs from that of HBV (99). HSPG and the low-density lipoprotein receptor (LDLR) play critical roles in HCV entry. Once the RNA genome is released, it is replicated and translated in the rough endoplasmic reticulum, where it is into 10 different proteins. These proteins are assembled and matured in the Golgi apparatus and then released to infect other cells. The involvement of LDLR and HSPG in HCV entry (105), which is also observed in the HBV viral cycle, is noteworthy and should be emphasized.

*In vitro* studies of Lf against HCV are less up to date than those HBV and other viruses discussed. Nevertheless, the results are still significant enough to suggest the need for further research on HCV and Lf. For example, an *in vitro* study examined the antiviral activity of hLf on HCV in Huh-7 cells (32). The first experiment in the study revealed that hLf treatment significantly decreased HCV replication in the cells. A notable finding during the study was the increased intracellular levels of hLf in the infected cells, indicating that the protein was taken up from the extracellular environment. This observation led to the conclusion that the neutralization activity of hLf likely occurs inside the host cell. The following experiments were conducted to evaluate the efficacy of hLf on HCV viral enzymes, where hLf demonstrated high efficacy against helicase/ATPase, inhibiting the activity of these enzymes and potentially preventing HCV infection. Similar studies have also reported the intracellular activity of different types of Lfs against HCV (47).

A study investigated the antiviral activity of camel Lf-derived peptide (cLf36) against HCV in an *in vitro* experiment (106). Three different concentrations of the peptide were tested: 44, 88, and 176 µg/mL. The 44 µg/mL concentration did not show a significant effect against HCV, while higher concentrations demonstrated notable antiviral activity. The molecular docking analysis revealed an interaction between the cLf36 peptide and E2 protein of HCV, suggesting that the peptide could inhibit viral entry and intracellular replication of HCV. The same peptide, cLf36, was also compared with new generation drugs against HCV (107). The binding strength of cLf36 to HCV was found to be 99.92, significantly higher than the 61.54 binding strength of the drugs tested, indicating the cLf36 might have superior antiviral potential. Considering the significant antimicrobial activity of camel Lf, camel Lf-derived peptides could be promising candidates for further investigation in antiviral studies.

Another study emphasized the significant antiviral activity of N-terminal region of camel Lf against HCV *in vitro* (46). Both the N-terminal and C-terminal regions of camel Lf were used against HCV, along with the full-length camel Lf. The N-terminal region demonstrated the highest prevention of HCV entry at 76%, compared to29% for the C-terminal and 69.39% for the full-length camel Lf. In terms of the inhibiting intracellular HCV replication, camel Lf achieved 100% inhibition at the highest concentration. The C-terminal region showed complete inhibition (100%) only at the highest concentration during the second treatment, whereas the N-terminal region prevented intracellular replication by 99% even at the lowest concentration tested. These findings show the potential of Lf-derived peptides against HCV and highlight the significance of N-terminal region in antiviral application.

The antiviral activity of Lf against hepatitis viruses has been intensively studied in recent years. However, while the current literature suggest some potential, recent research on Lf and hepatitis remains limited. The relationship between Lf as a lactic glycoprotein and the mother to child transmission potential of HBV is particularly interesting area that has only started to be explored. It is evident that earlier findings need to be updated, and future studies should provide more comprehensive data. Given the different genome structures of HBV and HCV, the potential role of Lf in both types of hepatitis could represent a promising focus for new research efforts.

## 5 Conclusions and further perspectives

The antiviral activity of Lf affects various types of viruses, including those causing infectious diseases. Existing studies on Lf’s antiviral activity highlight several key mechanisms: disrupting viral entry by competing for receptor binding, direct interaction with viral particles, and initiating immune responses through Lf-immune cell interaction. These mechanisms demonstrate the multifunctionality of Lf against viruses, which is a desired feature for antiviral treatments. However, current literature lacks sufficient comprehensive reviews and studies to verify most of these mechanisms. For example, flaviviruses can infect humans at high levels and pose a significant global threat. While there has been an increase in research on Lf, most studies are limited in diversity and do not clearly explain the molecular mechanisms involved.

Nevertheless, many studies have shown that Lf interacts with anionic surface molecules of host cells and viruses. The cationic structure of Lf and the anionic structure of GAG create a natural interaction that induces the antiviral activity, making Lf a competitor for the virus’s binding sites during the infection phase. However, certain limitations of Lf’s mechanisms need to be considered for future studies. As discussed in this review, Lf has different efficiencies against specific viruses, such as Zika and Chikungunya viruses, depending on the infection during Lf administration. The study revealed that Lf inhibited both viruses when administered during the infection phase, potentially indicating significant receptor competition. However, certain studies suggest that the Zika virus does not dependent on HSPG receptors to initiate viral entry. Based on this, the antiviral results should not attribute the inhibition of the Zika virus to receptor competition unless Lf interferes with other receptors involved in the viral entry of Zika virus. If not, Lf may inhibit the Zika virus through a different approach, potentially an intracellular mechanism, as suggested by other studies in the literature. This natural interaction is also observed with the cellular components of virus-infected cells. Many studies have shown that Lf can bind directly to viral particles and significantly prevent viral infection. In cases where receptor affinity is ineffective, this mechanism constitutes the antiviral activity of Lf. The current understanding of potential intracellular mechanisms involving Lf is insufficient to draw acceptable conclusions. However, some studies suggest potential intracellular activity of Lf, such as modulating specific pathways to reduce apoptosis or increase cell survivability, inhibiting enzymes essential for viral replication, or targeting the RNA/DNA within infected cells. Moreover, Lf is known for its immunomodulatory activity, promoting the synthesis and function of certain immune cells, along with regulating excessive cytokine levels during viral infections. Thanks to this properties, Lf’s role in viral infections is not limited to direct interaction; it can also modulate the immune system and the host response, potentially enhancing its antiviral activity when direct mechanisms are less effective.

The existing literature indicated that Lf has significant potential for unique research in antiviral therapy. Its antiviral activity is influenced by various parameters of the infected cell and the virus. Identifying which parameter affects which mechanism and to what extent would be the key to developing efficient, virus-specific antiviral treatments including Lf. Furthermore, new findings could provide insights into viral infections by revealing specific virus-cell interactions, thereby facilitating the development of more efficient antiviral therapies in the future. Lf can play an important role in this investigation. However, some existing data need to be updated and extended to provide a more comprehensive basis for therapeutic development. Although Lf is a high valuable glycoprotein with considerable potential, much research is still needed to explore its practical applications fully.
